# Polymorphisms in *MTHFR, MS* and *CBS* Genes and Homocysteine Levels in a Pakistani Population

**DOI:** 10.1371/journal.pone.0033222

**Published:** 2012-03-21

**Authors:** Mohsin Yakub, Naushad Moti, Siddiqa Parveen, Bushra Chaudhry, Iqbal Azam, Mohammad Perwaiz Iqbal

**Affiliations:** 1 Department of Biological and Biomedical Sciences, Aga Khan University, Karachi, Pakistan; 2 Department of Community Health Sciences, Aga Khan University, Karachi, Pakistan; University of Illinois at Urbana-Champaign, United States of America

## Abstract

**Background:**

Hyperhomocysteinemia (>15 µmol/L) is highly prevalent in South Asian populations including Pakistan. In order to investigate the genetic determinants of this condition, we studied 6 polymorphisms in genes of 3 enzymes - methylenetetrahydrofolate reductase (*MTHFR*; C677T; A1298C), methionine synthase (*MS;* A2756G), cystathionine-β-synthase (*CBS*; T833C/844ins68, G919A) involved in homocysteine metabolism and investigated their interactions with nutritional and environmental factors in a Pakistani population.

**Methodology/Principal Findings:**

In a cross-sectional survey, 872 healthy adults (355 males and 517 females; age 18–60 years) were recruited from a low-income urban population in Karachi. Fasting venous blood was obtained and assessed for plasma/serum homocysteine; folate, vitamin B12, pyridoxal phosphate and blood lead. DNA was isolated and genotyping was performed by PCR-RFLP (restriction-fragment-length- polymorphism) based assays. The average changes in homocysteine levels for *MTHFR* 677CT and TT genotypes were positive [β(SE β), 2.01(0.63) and 16.19(1.8) µmol/L, respectively]. Contrary to *MTHFR* C677T polymorphism, the average changes in plasma homocysteine levels for *MS* 2756AG and GG variants were negative [β(SE β), −0.56(0.58) and −0.83(0.99) µmol/L, respectively]. The average change occurring for *CBS* 844ins68 heterozygous genotype (ancestral/insertion) was −1.88(0.81) µmol/L. The combined effect of *MTHFR* C677T, *MS* A2756G and *CBS* 844ins68 genotypes for plasma homocysteine levels was additive (p value <0.001). Odds of having hyperhomocysteinemia with *MTHFR* 677TT genotype was 10-fold compared to *MTHFR* 677CC genotype [OR (95%CI); 10.17(3.6–28.67)]. Protective effect towards hyperhomocysteinemia was observed with heterozygous (ancestral/insertion) genotype of *CBS* 844ins68 compared to homozygous ancestral type [OR (95% CI); 0.58 (0.34–0.99)]. Individuals with *MTHFR* 677CT or TT genotypes were at a greater risk of hyperhomocysteinemia in folate and vitamin B12 deficiencies and high blood lead (p value <0.05) level.

**Conclusions:**

Gene polymorphism (especially *MTHFR* C677T transition), folate and vitamin B12 deficiencies, male gender and high blood lead level appear to be contributing towards the development of hyperhomocysteinemia in a Pakistani population.

## Introduction

Cardiovascular disease (CVD) which is defined as diseases of the heart and blood vessels of the body, has reached epidemic proportions in South Asia [1]. Recent reports have shown that hyperhomocysteinemia (>15 µmol/L) - an established risk factor for CVD is highly prevalent in the Pakistani population [2]. While deficiency of folate and vitamin B12 have been found to be associated with high levels of plasma homocysteine, the role of polymorphisms in combination of genes of enzymes involved in homocysteine metabolism need to be explored in the South Asian general population. The 3 major enzymes involved in homocysteine metabolism include methylenetetrahydrofolate reductase (MTHFR), methionine synthase (MS) and cystathionine β synthase (CBS). Various studies carried out mostly in the West have shown that 677T *MTHFR* allele has been associated with increased levels of plasma homocysteine in ethnic Europeans [3], while a 68-bp insertion in *CBS* gene (844ins68) and A2756G transition of *MS* gene have been found to be associated with low levels of plasma homocysteine in a population in the Midwestern region of the USA [4].

A recent study carried out in Singapore indicates that nationals of Indian descent had the highest frequency of *CBS* 844ins68 allele and *MS* 2756G allele, while Singaporean Chinese had the highest frequency of *MTHFR* 677T allele [5]. This indicates that genetic determinants of plasma homocysteine in Asians vary to a great extent. The objective of the present study was to evaluate the prevalence estimates of C677T and A1298C polymorphisms in *MTHFR* gene, A2756G polymorphism in *MS* gene and T833C/844ins68 and G919A polymorphisms in *CBS* gene and their relationship with plasma levels of homocysteine in a Pakistani population. Another objective was to study the interactions of these polymorphisms with related nutritional and environmental factors.

## Methods

### Ethics Statement

The study had been approved by the Ethics Review Committee of the Aga Khan University. Prior written informed consent was obtained from all the study participants.

### Study participants

In this cross-sectional study, 872 healthy subjects, 355 males and 517 females of age 18–60 years, from a low income urban locality in Karachi were included. A systematic random sampling was adopted as described in a previous publication [6]. Healthy individuals were screened by community health workers who were trained to collect demographic and clinical information. The clinical examination was carried out by the general physician. The criteria used to ensure that the participants were healthy have been described in a previous paper [2].

### Blood sampling and biomarkers assay

Ten mL of fasting venous blood was collected equally in heparinized and nonheparinized tubes. Whole blood was used for estimation of blood lead (Pb) [6], while plasma/serum was used for estimation of folate, vitamin B12 and pyridoxal phosphate (PLP; a coenzymic form of vitamin B6) using radioassays [7]–[9]. Plasma homocysteine was measured using immunoassay based kit (Abbott Laboratories Ltd; Pakistan).

### Genotyping of *MTHFR* C677T and A1298C polymorphisms

DNA was extracted from leukocytes according to the established protocols [10]. The polymorphisms C677T and A1298C in *MTHFR* were determined by polymerase chain reaction (PCR) followed by restriction fragment length polymorphisms (RFLP) [11]–[13]. The amplified fragments of C677T and A1298C polymorphisms were digested with restriction enzymes, *Hin*fI and *Mbo*II (New England Biolabs, USA), respectively and analyzed by gel electrophoresis.

### Genotyping of *MS* A2756G polymorphism

*MS* A2756G genotype was analyzed by PCR followed by RFLP as described by Matsuo et al. [12]. The amplified DNA fragment was digested with *Hae*III (New England Biolabs, USA) and electrophoresed in a 3% agarose gel.

### Genotyping of *CBS* 844ins68, T833C and G919A polymorphisms

The polymorphisms 844ins68, T833C and G919A in *CBS* gene were determined using PCR-RFLP based assays using restriction enzymes *Bsr*I (New England Biolabs, USA) for 844ins68 and T833C and *Alu*I (New England Biolabs, USA) for G919A [14]. Genotypes of *CBS* 844ins68 were defined as 68AA (ancestral homozygous) also reported in the literature as wild homozygous, 68AI (ancestral/insertion heterozygous) and 68II (insertion homozygous).

### Statistical analysis

All statistical analyses were done with the help of Statistical Package for Social Sciences® (SPSS) software version 13 for Windows® Apache Software Foundation, USA.

Mean (SD) were expressed for continuous variables such as homocysteine, folate, PLP and vitamin B12. One way ANOVA was used to assess the mean difference for the above mentioned continuous variables across genotypes, or combined genotypes. Bonferroni test for multiple pair-wise comparisons was used when the difference was found to be significant using ANOVA. Independent sample t-test was also used to assess the mean difference of above mentioned biomarkers. Regarding *CBS* 844ins68 genotype, only three participants were homozygous for the mutated gene (II), therefore, they were excluded from analysis. To test for interaction between the *CBS* and *MS* genotypes, a 2-way ANOVA model was carried out, however a 3-way ANOVA model was used to examine the interaction between the *CBS*, *MS* and *MTHFR* genotypes and to find out which of the polymorphisms were associated with plasma homocysteine levels after controlling for other genes. Multiple linear regression was used to obtain the relationship between homocysteine level and polymorphisms in three genes. Multiple coefficient of determination (R^2^) describes the variation in homocysteine levels as a consequence of combination of polymorphisms.

Binary logistic regression analysis was used to examine the association between risk of hyperhomocysteinemia (dependent variable) and genotypes examined in the study population (independent variable). Similar analyses were done to assess risk of hyperhomocysteinemia with combination of genotypes, and genotype-nutrient and genotype-blood Pb relationships. These models were also adjusted for age, gender, folate and vitamin B12 to assess the risk of hyperhomocysteinemia. Similarly, multiple linear regression was used to assess the average change in homocysteine levels while adjusting for gender, folate deficiency, B12 deficiency, blood Pb and three gene polymorphisms (*MTHFR* C677T, *MS* A2756G and *CBS* 844ins68).

## Results

### Allele frequency, genotype percentage and plasma/serum concentration of biomarkers for SNPs

The distribution of allele frequencies and genotype percentage values for single nucleotide polymorphisms (SNPs), *MTHFR* C677T, *MTHFR* A1298C, *MS* A2756G, *CBS* 844ins68 have been given in Table 1. All the distributions were found to be in agreement with Hardy-Weinberg equilibrium.

**Table 1:** Allelic frequencies and genotype percent of single nucleotide (SNPs) in *MTHFR, MS* and *CBS* genes.

SNP	Allele Frequency		Genotype (%)			HWE p
***MTHFR***	C	T	CC	CT	TT	
C677T	0.85	0.15	71.3	26.1	2.5	0.83
***MTHFR***	A	C	AA	AC	CC	
A1298C	0.45	0.55	20.8	48.7	30.5	0.63
***MS***	A	G	AA	AG	GG	
A2756G	0.71	0.29	52.4	38.8	8.8	0.20
***CBS***	A	I	AA	AI	II	
844*ins*68	0.93	0.07	86.5	13.2	0.34	0.52

Table shows Hardy-Weinberg equilibrium (HWE) testing. It indicates that the subjects were found to follow HWE. This analysis gives the p value and there is no deviation from the expected population structure. In the above four markers population stratification did not exist. “AA” refers to the ancestral/ancestral homozygous; while “AI” and “II” indicate ancestral/insertion heterozygous variant and insertion homozygous variant, respectively.

With the exception of a few reports [15], most studies have shown that *CBS* T833C co-segregates in *cis* with *CBS* 844ins68 insertion [16]. We also found that *CBS* T833C transition was co-segregating in *cis* with *CBS* 844ins68 in all individuals. Therefore, in analysis, we focused only on *CBS* 844ins68 polymorphism rather than *CBS* T833C. Genotyping of *CBS* G919A polymorphism showed that all the individuals had GG genotype, and therefore were not included in further analyses. The prevalence estimates of heterozygous CT and homozygous TT variants of *MTHFR* 677 polymorphism were found to be 26.1% and 2.5%, respectively. The heterozygous “AI” insertion prevalence was 13.2% which appears to be relatively high compared to previous reports from Asian populations [17]. The frequency of homozygous “II” insertion was 0.34%.

The distribution of circulating plasma/serum concentration of biomarkers, homocysteine, folate, PLP and vitamin B12 with respect to various genotypes of the above mentioned 3 genes has been summarized in Table 2. The mean homocysteine concentration was higher in individuals who were either heterozygous or homozygous variant for *MTHFR* C677T polymorphism. Bonferroni pair wise comparison revealed that plasma homocysteine value in TT genotype was significantly different from CC and CT genotypes (p value <0.001 and p value = 0.04, respectively). It was also observed that mean serum folate levels were significantly less in *MTHFR* TT genotype with respect to other genotypes (CC and TT) of *MTHFR* 677 (p value = 0.004). Mean homocysteine concentration was significantly lower in individuals carrying the *CBS* 844ins68 insertion compared to those without the insertion (p value = 0.01). Mean folate concentration was also significantly higher in subjects carrying the insertion as compared to those who were without insertion (p value = 0.01). We did not observe any significant differences among other biomarkers and SNPs of two genes, *MTHFR* A1298C and*MS* A2756G.

**Table 2:** Circulating concentrations of biomarkers with respect to various genotypes.

SNP	Plasma Homocysteine (µmol/L)	Serum Folate (ng/mL)	Plasma PLP (nmol/L)	Serum B12 (pg/mL)
***MTHFR*** ** 677**				
CC (n = 622)	13.97(7.62)	6.90(4.56)	33.14(33.50)	431(215)
CT (n = 228)	15.66(10.81)	6.00(4.34)	33.32(32.02)	466(262)
TT (n = 22)	29.32(23.57)	4.68(3.83)	29.99(22.19)	453(252)
p value*	<0.001	0.004	0.90	0.13
***MTHFR*** ** 1298**				
AA (n = 181)	15.25(10.1)	6.48(4.66)	33.09(28.81)	407(174)
AC (n = 425)	14.35(8.17)	6.64(4.48)	32.66(30.89)	452(234)
CC (n = 266)	15.01(11.08)	6.64(4.45)	33.84(38.15)	445(251)
p value	0.35	0.9	0.9	0.08
***MS*** ** 2756**				
AA (n = 457)	15.03(10.29)	6.61(4.49)	35.18(40.44)	436(219)
AG (n = 338)	14.61(8.90)	6.57(4.42)	29.87(17.54)	454(233)
GG (n = 77)	14.23(7.70)	6.73(5.04)	35.01(33.41)	409(268)
p value	0.71	0.95	0.06	0.23
***CBS*** ** 844ins68** **				
AA (n = 754)	15.13(9.75)	6.46(4.45)	33.31(33.51)	441(232)
AI (n = 115)	12.66(8.01)	7.60(4.81)	32.04(28.68)	434(212)
p value	0.01	0.01	0.82	0.84

Values are expressed as mean (SD).

* p value was based on ANOVA.

** *CBS* 844ins68, AA: ancestral homozygous, AI: ancestral/insertion heterozygous.

We assessed the risk of hyperhomocysteinemia with *MTHFR*, *MS* and *CBS* genotypes. Compared to the ancestral type *MTHFR* 677 genotype “CC”, the odds of having hyperhomocysteinemia with homozygous “TT” variant was 10.17 (p value <0.001) adjusted for age, gender, folate and vitamin B12. We also observed protective effect towards hyperhomocysteinemia with heterozygous genotype of *CBS* 844ins68 when compared to the ancestral type *CBS* 844ins68 [OR (95% CI); 0.58 (0.34–0.99)]. We did not find any significant risk of hyperhomocysteinemia with *MTHFR* A1298C, *MS* A2756G genotypes in the study population (Table 3).

**Table 3:** Crude and adjusted Odds Ratios (OR) with 95% CI for hyperhomocysteinemia^1^ by genotype of different single nucleotide polymorphisms (SNPs) in *MTHFR, MS* and *CBS* genes.

SNP	Ancestral	Heterozygous variant	Homozygous variant
***MTHFR*** ** C677T**			
*Crude* 2	1	1.22 (0.88–1.69)	5.06 (2.03–12.61)*
*Adjusted* 3	1	1.48 (1.0–2.18)	10.17 (3.6–28.67)**
***MTHFR*** ** A1298C**			
*Crude* 2	1	0.73 (0.50–1.05)	0.71 (0.48–1.06)
*Adjusted* 3	1	0.67 (0.40–1.02)	0.92 (0.57–1.47)
***MS*** ** A2756G**			
*Crude* 2	1	1.0 (0.74–1.35)	0.84 (0.49–1.43)
*Adjusted* 3	1	0.92 (0.65–1.31)	0.68 (0.36–1.28)
***CBS*** ** 844** ***ins*** **68**			
*Crude* 2	1	0.58 (0.36–0.92)*	-
*Adjusted* 3	1	0.58 (0.34–0.99)*	-

1 Plasma homocysteine >15 µmol/L.

2 Values are OR (95% CI) from logistic regression, *p value <0.05.

3 Values are OR (95% CI) from logistic regression adjusted for age (years), gender, folate and vitamin B 12.

* p value <0.05,

** p value <0.001.

The average changes in homocysteine levels for *MTHFR* 677CT and TT genotypes were positive [β (SE β) 2.01(0.63) and 16.19(2.00) µmol/L; p value = 0.001 and p value <0.001], respectively (Table 4). Contrary to *MTHFR* 677 polymorphism, the average changes in plasma homocysteine levels for *MS* 2756AG and GG variants were negative [β (SE β) −0.56(0.58) and −0.83(0.99) µmol/L; p value = 0.33 and p value = 0.4, respectively]. The average change occurring with *CBS* 844ins68 heterozygous variant was −1.88(0.81) µmol/L, (p value = 0.02). We have also observed increased levels of homocysteine for male gender, folate deficiency and B12 deficiency (Table 4).

**Table 4:** Crude and adjusted regression coefficients with 95% CI for the predictors of homocysteine levels (µmol/L) in Pakistani adults.

Factors	Crude		Adjusted*	
	(*n* = 872)		(*n* = 872)	
	β (SE β)	p value	β (SE β)	p value
**Males**	8.27 (0.56)	<0.001	8.27 (0.56)	<0.001
**Folate deficiency** **	4.41 (0.71)	<0.001	2.61 (0.62)	<0.001
**B12 deficiency** ***	3.87 (1.08)	<0.001	3.26 (0.92)	<0.001
**Blood Pb (>10 µg/dL)**	1.35 (0.65)	0.04	0.63 (0.55)	0.25
***MTHFR*** ** 677 CT**	1.17 (0.73)	0.11	2.01 (0.63)	0.001
***MTHFR*** ** 677 TT**	14.90 (2.00)	<0.001	16.19 (1.8)	<0.001
***MS*** ** 2756 AG**	−0.31 (0.66)	0.64	−0.56 (0.58)	0.33
***MS*** ** 2756 GG**	−0.62 (1.14)	0.58	−0.83 (0.99)	0.40
***CBS*** ** 844ins68 AI**	−2.44 (0.94)	0.01	−1.88 (0.81)	0.02

* Adjusted for variables in table.

** Folate levels ≤3.5 ng/mL.

*** B12 levels ≤200 pg/mL.

AI = ancestral/insertion (heterozygous).

### Gene-gene interaction

The combined effect of *CBS* 844ins68 heterozygous variant and the *MS* G2756 allele in lowering plasma homocysteine concentrations was also assessed. The mean plasma homocysteine concentrations in individuals who were carriers of both variants [844ins68 AI; 2756 AG; 11.4(4.9) µmol/L] appeared to be lower compared to carriers of either variant [844ins68 AA; 2756 AG; 15.14(9.29) µmol/L or 844ins68 AI; 2756 AA; 13.13(9.29) µmol/L]. To investigate the combined effect of these two genotypes in lowering the homocysteine levels, we applied 2-way ANOVA, and found that there was no significant interaction between the 844ins68 variant of the *CBS* gene and the A2756G transition of the *MS* gene for plasma homocysteine (p value = 0.26). However, using a 3-way ANOVA model we observed that the combined effect of *MTHFR* C677T, *MS* A2756G and *CBS* 844ins68 genotypes for plasma homocysteine was additive (p value <0.001), though interactions between variants of these three genes for plasma homocysteine levels were not significant (p value = 0.75). The results of this ANOVA model also showed that C677T transition of the *MTHFR* and 844ins68 variant of *CBS* gene were significantly associated with plasma homocysteine (p value = 0.04 and p value = 0.003, respectively) after controlling for other genes. However, no significant association between plasma homocysteine and A2756G transition of the *MS* gene was observed (p value = 0.165). The variation in plasma homocysteine because of the combined effect of these three genotypes was found to be 8.4%.

We hypothesized that risk associated between *MTHFR* C677T genotypes and hyperhomocysteinemia may be modified by variation in genes coding for other homocysteine metabolizing enzymes. It was observed that individuals having *MTHFR* 677 TT variant along with *MTHFR* 1298 AC variant had increased risk of hyperhomocysteinemia (OR, 8.78; Table 5). *MTHFR* 677 TT variant along with *MS* 2756 AA genotype, and *MTHFR* 677 TT variant along with *CBS* 844ins68 AA genotype also showed significantly increased risk for hyperhomocysteinemia, (ORs, 19.1 and 14, respectively). We did not find any increased risk associated with combined effect of *MTHFR* A1298C with *MS* A2756G and *CBS* 844ins68 in relation to hyperhomocysteinemia. Similarly, non significant relationship was observed between *MS* A2756G with *CBS* 844ins68 genotypes in relation to hyperhomocysteinemia (data not shown).

**Table 5:** Association of *MTHFR* C677T genotypes with genotypes of *MTHFR* A1298C, *MS* A2756G and *CBS* 844*ins*68 towards the risk of hyperhomocysteinemia^1^.

	*MTHFR* 677^2^		
	CC	CT	TT
	OR (95% CI)	OR (95% CI)	OR (95% CI)
***MTHFR*** ** 1298**			
AA	1.0 (reference)	1.40 (0.48–4.0)	3.99 (0.51–31.27)
AC	0.60 (0.37–0.96)*	0.96 (0.53–1.74)	8.78 (2.57–29.97)*
CC	0.68 (0.39–1.21)	1.10 (0.58–2.11)	00 N/A
***MS*** ** 2756**			
AA	1.0 (reference)	1.51 (0.89–2.56)	19.1 (4.61–79.0)**
AG	1.04 (0.68–1.59)	1.30 (0.70–2.39)	2.59 (0.45–14.85)
GG	0.58 (0.27–1.24)	1.64 (0.52–5.14)	00 N/A
***CBS*** ** 844** ***ins*** **68**			
AA	1.0 (reference)	1.39 (0.91–2.1)	14.0 (4.02–8.97)**
AI	0.47 (0.24–0.90)	1.35 (0.52–3.44)	3.87 (0.33–41.68)
II	00 N/A	00 N/A	00 N/A

1 Plasma homocysteine >15 µmol/L.

2 Values are OR (95% CI) from logistic regression adjusted for age (year), gender, folate and vitamin B12.

* p value <0.05;

** p value <0.001.

AA: Ancestral homozygous, AI: Ancestral/insertion, II: insertion/insertion.

### Gene-nutrient interaction

Since study participants had high prevalence of folate and vitamin B12 deficiencies (27.5% and 9.74%, respectively), these deficiencies were found to increase the risk of hyperhomocysteinemia (2.5 times with folate deficiency and 2.6 times with vitamin deficiency) in this population [2]. We, hypothesized that association between *MTHFR* 677 TT genotype and hyperhomocysteinemia could be modified by the folate and vitamin B12 status of individuals in this study population. We observed that the risk of hyperhomocysteinemia increased with *MTHFR* C677T and TT genotypes with folate and vitamin B12 deficiency states (Table 6). As we found only 22 individuals with the TT genotype, heterozygote and homozygote mutant individuals were combined for the above analysis.

**Table 6:** Association of *MTHFR* C677T genotypes with folate and vitamin B12 deficiency states and risk of hyperhomocysteinemia^1^.

	*MTHFR* 677^2^	
	CC	CT or TT
	OR (95% CI)	OR (95% CI)
**Folate**		
Normal (>3.5 ng/mL)	1.0 (reference)	1.48 (0.93–2.34)
	*n* (469)	*n* (163)
Deficiency (≤3.5 ng/mL)	1.99 (1.29–3.08)*	4.84 (2.80–8.37)**
	*n* (153)	*n* (87)
**Vitamin B12**		
Normal (>200 pg/mL)	1.0 (reference)	1.96 (1.33–2.88)*
	*n* (563)	*n* (224)
Deficiency (≤200 pg/mL)	2.68(1.41–5.07)*	4.33 (1.7–10.86)*
	*n* (59)	*n* (26)

1 Plasma homocysteine >15 µmol/L.

2 Values are OR (95% CI) from logistic regression adjusted for age (year) and gender.

* p value <0.05;

** p value <0.001.

### Gene – environment interaction

Fifty nine percent of the study participants had mean (SD) blood Pb levels [11.65(5.5) µg/dL] above the previously considered acceptable levels (10 µg/dL) and, therefore would pose high risk for hyperhomocysteinemia in the study subjects [6]. Thus, association statistics for gene-environment interaction was carried out to study the relationship between *MTHFR* C677T genotypes and blood Pb concentration toward the risk of hyperhomocysteinemia. It turned out that individuals with either heterozygous “CT” or homozygous “TT” variant genotype of *MTHFR* 677 who also had blood Pb levels greater than 10 µg/dL, had significantly increased risk for hyperhomocysteinemia [OR (95% CI) 2.36 (1.42–3.9); p value = 0.001] compared with those having “CC” genotype of *MTHFR* 677 with blood Pb levels less than or equal to 10 µg/dL, even when the model was adjusted for age, gender, folate and vitamin B12 (Table 7). To the best of our knowledge, the relationship of the blood Pb concentration with *MTHFR* C677T genotypes for hyperhomocysteinemia has never been reported.

**Table 7:** Association of *MTHFR* C677T genotypes with blood Pb levels towards risk of hyperhomocysteinemia^1^.

	*MTHFR* 677^2^	
	CC	CT or TT
	OR (95% CI)	OR (95% CI)
**Blood Pb**		
(≤10 µg/dL)	1.0 (reference)	1.36 (0.75–2.45)
	*n* (263)	*n* (97)
(>10 µg/dL)	1.15 (0.76–1.73)	2.36 (1.42–3.90)*
	*n* (359)	*n* (153)

1 Plasma homocysteine >15 µmol/L.

2 Values are OR (95% CI) from logistic regression adjusted for age (year), gender, folate and vitamin B12.

* p value <0.05.

### Gene-gene, gene-nutrient and gene-environment combined influence on plasma homocysteine

In order to evaluate the magnitude of change in plasma homocysteine by various factors, multiple linear regressions were used while adjusting for covariates. It turns out that male gender, folate deficiency, B12 deficiency and *MTHFR* 677 polymorphisms have significant positive association with plasma homocysteine. Whereas *CBS* 844ins68 polymorphism had significant negative influence on plasma homocysteine levels after adjusting for other covariates (Table 4). The overall combined effect of gender, status of folate, status of vitamin B12, blood Pb levels and genotypes of the three genes (*MTHFR, MS* and *CBS*) on plasma levels of homocysteine through multiple linear regression model, revealed that 30% of plasma homocysteine in the study population was contributed by the above mentioned factors.

## Discussion

Homocysteine metabolism pathway has a pivotal link with “one-carbon metabolism”. Functional polymorphisms in genes encoding major enzymes in one-carbon metabolism, *MTHFR* C677T, *MS* A2756G, methionine synthase reductase (*MTRR* A66G) and thymidylate synthase (*TS*) will have profound effect on folate metabolism and hence influence intracellular methylation reactions [18]. The resulting hyperhomocysteinemia is one of the indicators of impaired methylation capacity and could lead to a number of pathological processes including atherosclerosis.

Previous reports suggest that low levels of folate, vitamin B6 and vitamin B12 are associated with increased level of plasma homocysteine [3], [19]; yet, this association is not absolute, suggesting that environmental and genetic factors could also be playing a role. A majority of studies on genetic regulation of plasma homocysteine levels have been confined to single nucleotide polymorphism (C677T) of the *MTHFR* gene. Moreover, only a few studies have been carried out on the Pakistani general population addressing association of plasma homocysteine with polymorphisms other than the *MTHFR* gene. We analyzed six SNPs in enzymes involved in homocysteine metabolism (i.e., *MTHFR* C677T and A1298C; *CBS* T833C, G919A and 844ins68, and *MS* A2756G) in 872 healthy study subjects and studied their influence on the risk of hyperhomocysteinemia in this population.

The present findings regarding allelic frequencies of 0.85 and 0.15 for *MTHFR* 677C and 677T alleles, respectively, are in line with the reports published on Pakistanis and Asian Indians [19], [20]. Moreover, 115 individuals out of 872 with T833C/844ins68 polymorphism reveal a relatively higher percentage of heterozygotes (13.2%) of *CBS* gene as compared to prevalence of heterozygotes among the Northern Chinese population [17]. However, these values are lower than those quoted for the Spanish and Sub Saharan populations [21] but quite comparable to those found in the Turkish, Iranian and Indian populations [22]–[24]. In the present investigation, we have also observed consistent co-segregation of these two polymorphisms in *cis*, which is well supported by previous observations [16], [25], [26]. The frequency of homozygous “II” insertion genotype was 0.34% which compares well with 0.2% in a Russian population [27] and 0.6% in a Thai population [28]. There have been conflicting reports about the role of *CBS* G919A transition in different populations. While 919A allele is highly prevalent in the Irish homocystinuria population [29], it was conspicuously absent in the populations in Italy, The Netherlands, Germany, Czech Republic, USA and Brazil [4], [26], [30]. Our reason for investigating this transition in the Pakistani population was also based on the fact that 919A allele was found to be associated with decreased risk of coronary heart disease in a Chinese population [31]. However, complete absence of 919A allele in our study population is suggestive that there is no significant role of *CBS* G919A transition in modulating homocysteine metabolism in the Pakistani population. Similarly, no individual was found to have *CBS* T833C transition without insert fragment.

Our findings corroborate previous results that individuals with *MTHFR* 677T allele are more likely to have elevated plasma homocysteine [4], [16]–[32]. In the *MS* gene, A2756G transition is one of the most common polymorphisms reported in the literature in the context of plasma homocysteine [4]. We report that approximately 48% of the individuals living in this community of Karachi are either heterozygous or homozygous carriers of 2756G allele.

Based on the observations that 68 bp insertion of the *CBS* gene and the A2756G transition of the *MS* gene were associated with decreased concentrations of plasma homocysteine, whereas, C677T transition of the *MTHFR* gene tended to increase plasma homocysteine levels, the 3-way ANOVA showed that the effect of all three polymorphisms appeared to be additive as the model showed no interaction between the three variants (p value = 0.075)). Thirteen percent of the individuals in our study population were carriers of the 68 bp insertion of the *CBS* gene, 48% were either heterozygous or homozygous carriers of the G2756 allele of the *MS* gene and 28% were either heterozygous or homozygous variants for the C677T polymorphism of the *MTHFR* gene. This indicates that over half of the individuals' genotype (*CBS* gene and *MS* gene) were modulating the effect of *MTHFR* C677T polymorphism on plasma homocysteine levels. However, the net effect of these genotypes appeared relatively moderate as the combined overall variation in plasma homocysteine was found to be 8.4% from the normal concentration.

Our results also suggested that *MTHFR* 677TT genotype along with *MTHFR* 1298 AC genotype was associated with increased risk of hyperhomocysteinemia. Similar increased risk was observed with combinations of *MTHFR* 677 TT and *MS* 2756 AA genotypes and *MTHFR* TT and *CBS* 844*ins*68 AA genotypes. Since prevalence of TT genotype is relatively low in the Pakistani population (2–3%), it is conceivable that *MTHFR* dimorphism may not be having a major role in causing hyperhomocysteinemia in this population [19], [33]. Moreover, high prevalence of 2756 G variant of *MS* gene and modestly increased AI variant of *CBS* 844ins68 gene (observed in the present study and both of them having homocysteine lowering effect) might be neutralizing the homocysteine increasing effect of TT variant. This is further supported by decrease in the risk of hyperhomocysteinemia in various combinations of *MTHFR* 677TT with *MS* 2756AG variant and *CBS* 844ins68 AI variant (Table 5).

Interaction between B vitamin deficiencies and defects in genes regulating homocysteine metabolism has also been reported previously for the C677T polymorphism of the *MTHFR* gene [34]. We have observed that individuals with either CT or TT variant of *MTHFR* 677 along with folate deficiency have nearly 5 times increased risk of hyperhomocysteinemia, while risk of hyperhomocysteinemia increases 4.33 fold in individuals carrying CT or TT variant of *MTHFR* 677 with vitamin B 12 deficiency.

Association between *MTHFR* C677T genotypes and blood Pb levels is a novel finding of the present study. We have observed that individuals having 677T allele of the *MTHFR* gene with high blood Pb levels have 2.36 times increased risk of hyperhomocysteinemia. How individuals with 677T allele could be more susceptible to having high blood Pb and hyperhomocysteinemia merits some discussion. The enzyme MTHFR has appreciable number of thiol containing amino acids-12 methionine and 11 cysteine residues [35]. It is plausible that introduction of valine (an amino acid with nonpolar side chain) in place of threonine (an amino acid with polar side chain) in the variant enzyme might be making these thiol amino acids more accessible to binding by Pb. If binding of Pb is at or near the active site of the enzyme, it might lead to impairment of enzyme function, hence decreased methylation of homocysteine to form methionine.

This study had several strengths, including a relatively large sample size, multiple SNPs and focus on gene-gene, gene-environment and gene-nutrient interactions. The association between combined *MTHFR* CT and TT genotypes and folate and B12 deficiency states indicates that decreased folate or cobalamin levels and MTHFR thermolibility due to T allele are intimately involved in the development of hyperhomocysteinemia. Moreover, high plasma homocysteine levels especially in CT and TT variants of *MTHFR* 677 in the presence of increased levels of blood Pb (>10 µg/dL) could be due to reduced plasma clearance of this amino acid because of chelation of Pb with homocysteine [36]. This is suggestive that exposure to Pb especially in individuals with 677T allele could be another factor for hyperhomocysteinemia in the Pakistani population.

This study also has some limitations. Although this is the largest and most comprehensive study on hyperhomocysteinemia at the community level in Pakistan, yet it includes only six variants in three genes involved in homocysteine metabolism and only one of them (*MTHFR* 677 TT) showed a significant positive association with plasma homocysteine. Since this variant has low prevalence in the Pakistani population [19], and the combined effect of 3 polymorphisms (*MTHFR* C677T, *MS* A2756G, *CBS* 844ins68) is 8.4%, it appears that additional variations in the genes involved in homocysteine metabolism (directly or indirectly) could be playing an important role in this population. For instance, variations in genes involved in the transport and/or absorption of folate, vitamin B6 or vitamin B12 could be highly prevalent. In a recent study, choline dehydrogenase (*CHDH* A119C) has been found to be significantly associated with plasma homocysteine in an Indian population [37]. Since liver has a major role in homocysteine metabolism, this polymorphism could be playing a significant role in maintaining homocysteine levels in circulation. Moreover, polymorphisms investigated in this study could be further examined for their role in increasing the risk towards development of CVD, which is highly prevalent in the Pakistani population [1]. Besides, genes coding for other enzymes of one-carbon metabolism that might be relevant to homocysteine homeostasis could be of significance in this population. Therefore, we believe that a genome-wide approach using linkage and association studies in large family cohorts, and large case-control studies could be a powerful tool in the detection of unknown polymorphisms associated with hyperhomocysteinemia in the Pakistani population.

In spite of these limitations, the study did provide evidence in support of the hypothesis that hyperhomocysteinemia is a complex multi-factorial condition, and in the Pakistani population genetic factors along with nutritional and environmental factors appear to play a role in the development of high levels of plasma homocysteine.
